# Exploring Risk Factors of Unexpected Death, Using Palliative Care Outcomes Collaboration (PCOC) Measures, among Terminal Patients Receiving Palliative Care in Taiwan

**DOI:** 10.3390/ijerph192013294

**Published:** 2022-10-15

**Authors:** Wen-Hsuan Hsiao, Chun-Li Wang, Lung-Chun Lee, Szu-Pei Chien, Chin-Chu Hsu, Wei-Min Chu

**Affiliations:** 1 Department of Medical Education, Taichung Veterans General Hospital, Taichung 407, Taiwan; 2 Department of Family Medicine, Taichung Veterans General Hospital, Taichung 407, Taiwan; 3 Institute of Medicine, Chung Shan Medical University, Taichung 402, Taiwan; 4 Department of Industrial Engineering and Enterprise Information, Tunghai University, Taichung 407, Taiwan; 5 School of Public Health, China Medical University, Taichung 404, Taiwan; 6 Department of Nursing, Taichung Veterans General Hospital, Taichung 407, Taiwan; 7 School of Medicine, National Yang-Ming Chiao Tung University, Taipei 112, Taiwan; 8 Department of Post-Baccalaureate Medicine, College of Medicine, National Chung Hsing University, Taichung 402, Taiwan; 9 School of Medicine, Chung Shan Medical University, Taichung 402, Taiwan; 10 Education and Innovation Center for Geriatrics and Gerontology, National Center for Geriatrics and Gerontology, Obu 474-8511, Japan

**Keywords:** unexpected death, palliative care, Palliative Care Outcomes Collaboration, end-of-life

## Abstract

Palliative care has the ability to relieve both physical discomfort and psychological distress in terminally ill patients. However, unexpected death may still occur in palliative care settings. This study aimed to utilize Palliative Care Outcomes Collaboration (PCOC) data to better determine any associated factors which may surround unexpected death in palliative care settings. Data were extracted from the PCOC database by the palliative care team within Taichung Veterans General Hospital (TCVGH). Data of deceased patients were extracted during the period from January 2021 to December 2021 from multiple palliative care settings. The deaths of patients whose last recorded palliative phase was 1–3 were defined as unexpected. A total of 280 deceased patients were included, with mean age at death being 67.73, 61% being male, and 83.2% cancer patients. We discovered that shortness of breath, as assessed by the Symptom Assessment Scale (SAS), decreased risk of unexpected death (OR: 0.91, 95% CI: 0.84–0.98), while impending death discharge (OR: 3.93, 95% CI: 1.20–12.94) and a higher Australia-modified Karnofsky performance status (AKPS) score (OR: 1.15, 95% CI: 1.10–1.21) were associated with unexpected death. Thus, medical staff must inform the family of patients early on regarding any risk factors surrounding unexpected death to help everyone involved be prepared in advance.

## 1. Introduction

Death is the inevitable result of life, with people and their family who are facing death often experiencing extreme amounts of stress, whether it be physical, psychological or spiritual aspects [1]. Nearly a half century ago, palliative care was first developed in the United Kingdom where it then spread to the rest of the world [2]. Evidence has shown that palliative care can help relieve the physical discomfort and psychological distress felt by terminally ill patients [3].

However, any sudden or unexpected death may still occur in palliative care settings, where an unexpected death refers to one which happened earlier than what was anticipated by the physician. From a previous study, sudden unexpected death occurs in a range of 6.4% to 16.8% in inpatient palliative care units [4]. Unexpected death disallows patients the opportunity to spend their remaining time as planned, while also causing significant and complicated grief to bereaved caregivers [5]. In recent studies, associated factors surrounding sudden death included male gender, older age, lung cancer, cancer with liver metastasis, malignant skin lesions, and symptoms of dyspnea and fatigue [4,6]. However, the associated factors regarding the unexpected death of patients receiving assistance from multiple palliative care services were never explored.

Thus, due to multiple negative encounters, an unexpected death is often regarded as being caused by a poor quality of care, particularly in palliative care settings [7]. There are multiple tools to assess the patients’ conditions, including support team assessment schedule (STAS) [8], palliative care outcome scale (POS) [9] and the gold standards framework (GSF) [6,10], and the clinicians can use the outcomes to adjust clinical practice and system, which in turn improves the quality of care. Recently, many countries have begun adopting the assessment framework of Palliative Care Outcomes Collaboration (PCOC) for quality improvement in palliative care.

The Palliative Care Outcomes Collaboration (PCOC) is a national system established in Australia to promote improvement in the quality of palliative care [11]. Five clinical assessment tools, including the Symptom Assessment Scale (SAS) [12], Palliative care problem severity score (PCPSS) [13], Australia-modified Karnofsky performance status (AKPS) [14], resource utilization groups–activities of daily living (RUG-ADL) [15], and the palliative care phase [16], have been used to assess a patient’s daily functional status and the symptomatic care of patients receiving palliative care. Through the long-term routine collection of point-of-care data, PCOC is able to measure the quality of palliative care services and benchmark the outcomes [11]. A certain number of hospitals in Taiwan implement PCOC, and the palliative care team within the Veterans Hospital medical system is part of it. 

The quality of death and dying in Taiwan has been ranked as the highest in Asia and has also, due to continuous improvement, progressed from being ranked sixth best in 2015 [17] to third in 2021 [18] worldwide. With regard to palliative care in Taiwan, the Department of Health has been dedicated to the development of hospice palliative care since the year 1995 [19]. In 1996, the National Health Insurance (NHI) plan began to include hospice home care in its funding program, while continuously offering hospice inpatient care, with the per capita and per diem program and palliative care consultation services, being subsidized, respectively, in 2000 and 2004 [20]. While these hospice care programs were initially only offered to terminal cancer patients, the NHI system finally expanded hospice care coverage to non-cancer terminally ill patients in 2009 [21], offering them palliative care specialist consultations and nursing care [22]. Regarding hospice palliative care policy, legislation entitled the “Hospice Palliative Care Act” in 2000, with amendments being added in both 2011 and 2013 [23]. Furthermore, “The Patient’s Right to Autonomy Act” was passed in 2015, which was the first special law to be enacted on patient autonomy in Asia [24]. 

To date, unexpected death amongst multiple palliative care services in Asia by Palliative Care Outcomes Collaboration (PCOC) assessment tools have not yet previously been well explored. This study aims to utilize PCOC data to determine the risk factors surrounding unexpected death in multiple palliative care settings.

## 2. Materials and Methods

Data were extracted from the PCOC database by the palliative care team within Taichung Veterans General Hospital (TCVGH). TCVGH houses more than 1500 beds; the largest public tertiary medical center in central Taiwan. The hospice palliative care team possesses the ability to provide comprehensive care because it is made up of various disciplines, including physicians, nurses, counseling psychologists, social workers, spiritual therapists, art therapists and trained volunteers. In addition, the scope of services is not only limited to our hospital, as it also expands to the outside community. 

PCOC assessment tools are utilized as a part of the daily routine clinical practice of TCVGH’s palliative care team by systematically recording each patient’s symptoms and medical concerns, as well as measuring the quality of care. Not only clinicians, but also patients, family and caregivers participate in the assessment. All of the clinicians who adopt PCOC assessment tools have attended PCOC workshops and sessions in order to ensure the quality and standards of all records.

We collected data on deceased patients during the period of January 2021 to December 2021 from multiple palliative care services, including the palliative care consultation service (PCCS), inpatient palliative care unit, and palliative home care service. PCOC data were recorded for all patients receiving palliative care services. For inpatient palliative care, PCOC data were recorded daily by registered nurses. For PCCS and palliative home care service, PCOC data were recorded when a palliative care physician/nurse visited the patient. 

PCOC clinical assessment tools included the Symptom Assessment Scale (SAS), Palliative care problem severity score (PCPSS), Australia-modified Karnofsky performance status (AKPS), resource utilization groups–activities of daily living (RUG-ADL), and palliative care phase. The SAS captured data that is subjective discomfort, including sleep disturbance, poor appetite, nausea/vomiting, gastrointestinal discomfort/constipation, shortness of breath, fatigue, pain and dry mouth. Each symptom was rated on a scale of 0 to 10 points, which was scored by the patients themselves or their caregivers [25]. The PCPSS data captured the problem severity in four palliative care domains, including pain, other physical symptoms, psycho-social-spiritual discomfort, family/caregiver problems. Each domain was rated on a scale of 0–3 points, and assessed by clinicians [13]. The AKPS scores captured each patient’s physical function, and rated from 0 (death)–100 (normal with no complaints, no evidence of disease) [14]. The RUG-ADL documents patients’ activities of daily living across 4 domains, including bed mobility, toileting, transfer, and eating. A three-point item (1–independent or supervision only; 3–total dependence/tube fed) was used in the eating domain, and the other three activities were based on five-point items (1–independent; 5–two or more persons assist) [15]. The Palliative Care Phase data recorded patients’ current general condition using 5 distinct phases: stable (phase 1), unstable (phase 2), deteriorating (phase 3), terminal (phase 4) and bereavement (phase 5) [16].

We defined unexpected deaths as the patients whose last recorded palliative care phase was 1–3. The analysis was performed to explore the risk factors of unexpected deaths. We collected multiple factors, including gender, types of palliative care services utilization, reasons for discharge, diagnosis, the presence of metastasis in cancer patients, duration of palliative care services received, and PCOC clinical assessment tools. 

Statistical analyses were performed using SAS version 9.4 (Statistical Analysis Software 9.4, SAS Institute Inc., Cary, NC, USA). Categorical variables, including gender distributions, types of palliative care services utilization, reasons for discharge, diagnosis, the presence of metastasis in cancer patients and PCPSS were all analyzed using chi-square tests. Continuous variables, including age distribution, duration of palliative care services received, SAS, RUG-ADL and AKPS were all analyzed through *t*-tests. Multivariate logistic regression was used to analyze risk factors surrounding unexpected death. A two-tailed *p* value < 0.05 was considered statistically significant.

From a previous study using AKPS as a level of function to define sudden death [26], we realized that a patient’s AKPS during their last days of life may be an important factor associated with unexpected death. Thus, subgroup analysis was performed to analyze if there were different risk factors among patients who had different AKPS during their last days of life. We grouped participants into two groups, according to their AKPS during their last week of life. One group included participants with at least one AKPS > 50 during their last week of life, while the other group involved participants with an AKPS < 50 during their last week of life. 

## 3. Results

Table 1 shows the characteristics of the 280 cases taken from the PCOC database. The mean age of the decedents at death was 67.7 years (SD 15.9) and 61% were male. Most decedents had cancer (83.2%) and had metastatic tumors (87.3%). Forty-six point nine percent (46.9%) of participants were under the care of PCCS, while 30.1% were cared for in an inpatient palliative care unit. For reasons of discharge, the proportion of death at hospital accounted for 86.1% of included patients. In addition, Table 1 shows the percentage of different distress types as evaluated by SAS and PCPSS, as well as the scores of AKPS and RUG-ADL. 

Table 2 presents whether there is any significant difference in characteristics between patients who experienced unexpected death and those who did not. The distribution of reasons for discharge, patients who experienced distress from fatigue, RUG-ADL, and pain and other symptoms of PCPSS were statistically different between patients with unexpected death and those without.

Univariate analyses were performed regarding the potential factors surrounding unexpected death, which were impending death discharge, feeling a shortness of breath, pain, AKPS and ADL scores (Table 3). In multivariable logistic regression, the results showed that after adjustments for age, gender, reasons for discharge, and PCOC assessments, which included SAS, AKPS, ADL and PCPSS, we discovered that feeling a shortness of breath on the SAS decreased risk of unexpected death (OR: 0.91, 95% CI: 0.84–0.98), while impending death discharge (OR: 3.93, 95% CI: 1.20–12.94) and a higher AKPS score (OR: 1.15, 95% CI: 1.10–1.21) were associated with unexpected death (Table 4). 

For subgroup analysis, the group of patients with AKPS scores <50 in the seven days prior to death who experienced distress from breathing (OR: 0.89, 95% CI: 0.80–0.99), poor appetite on the SAS (OR: 0.89, 95% CI: 0.80–0.99), or a higher RUG-ADL score (OR: 0.69, 95% CI: 0.57–0.83) were less likely to die unexpectedly, while having other symptoms of PCPSS (OR: 2.35, 95% CI: 1.16–4.76) was a risk factor for unexpected death. Alternatively, a higher RUG-ADL score (OR: 0.66, 95% CI: 0.44–0.99) also decreased risk of unexpected death in the group which had at least one dataset of AKPS scores >50 in the seven days prior to death (Table 5).

## 4. Discussion

In this study, we used the PCOC database involving deceased patients from multiple palliative care services, and defined unexpected death of those patients as those who died during the non-terminal phase. No significant differences in age, gender, model of palliative care services, diagnosis, the presence of metastasis in cancer patients or duration of palliative care services received were noted between patients who experienced expected death and those who died unexpectedly. The patients who suffered from breathlessness had a lower risk of unexpected death, while patients with an impending death discharge or a higher AKPS score had a higher risk of unexpected death.

Our results were different from those reported by two previous studies that explored breathlessness as an associated factor for unexpected death [4,6]. However, our results revealed that patients experiencing shortness of breath were less likely to die unexpectedly. This result may be due to the fact that multiple studies dedicated to identify predictors to evaluate terminal patients’ end-of-life status, and dyspnea at rest was one of the factors significantly associated with shortened survival [27]. Therefore, our clinicians were more aware of those symptoms, including poor performance status, appetite loss, edema, dyspnea at rest, delirium, etc [28]. Furthermore, we recorded the conditions of each patient every day, and that the patients had a higher chance of suffering from dyspnea as their conditions worsened, thus causing our clinicians to switch the patients to the terminal phase simultaneously. 

According to traditional Asian culture, a high proportion of the population wish to pass away peacefully at home in the company of relatives and friends. Therefore, impending death discharge (IDD) is unique yet common for dying patients in Taiwan [29]. In our study, impending death discharge led to a higher risk of unexpected death. One retrospective study performed in Taiwan explored the characteristics of palliative cancer patients with IDD, with the results indicating that patients at an older age, shorter hospice stay, gastrointestinal, peritoneal or pulmonary cancer tended to opt for IDD. By contrast, male patients experiencing oropharyngeal, bone, connective, breast or metastatic cancers were negatively correlated with IDD [30]. Terminal patients with IDD have relatively shorter hospice stays, therefore a palliative care provider may not be able to recognize all terminally ill symptoms or those patients at the terminal phase of PCOC. In addition, from one study in Canada evaluating factors associated with patients’ different discharge dispositions [31], we learned that there might be some associated factors of patients among different discharge dispositions. Some patients preferred IDD, but the unstable physical condition might make them unable to fulfill their choice, and passed away at hospital. Reason for discharge might be a result, but we thought patients who could discharge when impending death might have some associated factors, which caused unexpected deaths. Therefore, future studies remain warranted not only to better explore the early recognition of symptoms that may burden patients choosing IDD, but also to evaluate the associated factors about the patients with impending death discharge and the ones who chose IDD initially, but eventually died at hospital.

AKPS evaluates patient performances relating to work, daily activity and self-care, so patients with a higher AKPS score are those who are in relatively better health. Therefore, it is reasonable to conclude that a higher AKPS score contributes to unexpected death because a palliative care provider may regard patients with a better AKPS score as being relatively stable, while ignoring certain clues that may imply the patient is approaching their terminal phase of life. In a subgroup analysis of both groups, whether AKPS scores were <50 or >50 during the last week of life, a higher RUG-ADL score indicated a patient’s need for more care, which was negatively correlated to unexpected death. This suggested that patients who are given more attention by their healthcare professional have less chance of unexpected death. Thus, our results indicated that palliative care providers need to be aware of patients having better AKPS scores and fewer care needs.

As for patients with low AKPS scores, it is interesting to note that subjective symptoms seen from SAS, such as shortness of breath and poor appetite, decreased risk of unexpected death. However, objective symptoms were risk factors for an unexpected death. Concordance between self-reported symptoms and a professional’s assessment is a recently emerging topic. Simon et al. reported in a multi-center study that a professional’s assessment may be a valid alternative for breathlessness [32]. Future work and studies could focus on the factors surrounding the discordance between SAS and PCPSS from PCOC.

The definition of unexpected death or sudden death among terminally ill patients is another important issue to be discussed. Multiple studies have researched the associated factors surrounding unexpected death [4,6]. Although PCOC is commonly utilized in palliative care services in Australia, Ireland, and Taiwan [33], few studies have used PCOC data. An Australian study defined sudden death as a decline from an AKPS score of 50 or more to death in the last seven days of life, which meant if a patient had AKPS below 50 anytime during the last seven days of life, it was not sudden death as a definition. The average time of measurement was only once per patient per week before their death [6]. In our study, clinicians working in palliative care services evaluated PCOC scores every day, so most of the last AKPS scores before death were lower than 50, which could not meet the above definition of sudden death. Therefore, we defined unexpected death by the phase the patients were in, not only through their AKPS scores prior to death. However, subgroup analysis was performed which then made our results more comparable to previous studies.

Our study has several strengths. First, this is the first paper in Asia using PCOC assessment tools, which provide point-of-care data and are able to benchmark the outcomes. These tools are valuable for determining the risk factors surrounding unexpected death among deceased patients who had been receiving palliative care. Second, we compiled cases involving different models of palliative care services, not only in hospitals (palliative care consultation services and inpatient palliative care units), but also in palliative home care service, in order to better reveal real-world data. Third, we assessed the clinical conditions of patients and recorded PCOC scores every day. When comparing our study to previous studies, this approach brought our data closer to the actual condition of the patients prior to their death, while also being able to provide the most timely and appropriate response based upon the discomforts reported by the patients or caregiver, as well as our medical staff’s assessment.

There are some limitations to our study. First of all, our data were extracted from records of patients who died in a single hospital and were of the same nationality, which limits the study’s generalizability and may have selection bias. Second, interrater differences could occur because PCOC was possibly performed by different healthcare professionals each day. However, we attempted to avoid this possible bias by requiring all clinicians who performed PCOC assessments to attend PCOC workshops and sessions, thus ensuring the quality and standards of our records. Third, there were no relevant physiological data involved, such as blood examination or image reports in our PCOC database, so residual confounders could exist.

## 5. Conclusions

Our study results show that terminal patients facing impending discharge were at risk of unexpected death, while having shortness of breath or more care needs decreased risk of unexpected death. Palliative care professionals should ask and assess patients and their families with regard to the patient’s desired place to die, while also paying more attention to patient wishes surrounding IDD. Finally, medical staff must also inform the family of patients early regarding any risk factors surrounding unexpected death, in order to help everyone involved be prepared in advance.

## Figures and Tables

**Table 1:** Characteristics of 280 Deceased Patients in Multiple Palliative Care Service.

Characteristics	N/% Mean ± SD	
Age at death (years)	67.73 ± 15.88	
Gender		
Male	173	61.79%
Female	107	38.21%
Types of palliative care services utilization		
Inpatient palliative care unit	131	46.95%
Palliative care consultation service	84	30.11%
Palliative home care service	62	22.22%
General ward	2	0.72%
Missing data (n)	1	0.36%
Reasons of discharge		
Death	241	86.07%
Impending death discharge	39	13.93%
Diagnosis		
Cancer	233	83.21%
Non-cancer	47	16.79%
Metastatis present		
Yes	179	87.32%
No	26	12.68%
Missing data(n)	28	12.02%
Duration of PCOC ^1^ evaluation (days)	13.82 ± 19.70	
SAS ^2^		
Distress from difficulty sleeping	2.80 ± 3.10	
Distress from appetite	2.64 ± 3.33	
Distress from nausea	0.76 ± 1.85	
Distress from bowels	1.69 ± 2.76	
Distress from breathing	4.08 ± 3.35	
Distress from fatigue	3.94 ± 2.49	
Distress from pain	2.53 ± 2.91	
Total scores	18.44 ± 14.82	
Missing data	42	
AKPS ^3^	22.86 ± 12.11	
RUG-ADL ^4^	15.79 ± 2.20	
PCPSS ^5^		
Pain		
Continue care or monitor (0–1)	233	83.21%
Need further management (2–3)	47	16.79%
Other symptoms		
Continue care or monitor (0–1)	157	56.07%
Need further management (2–3)	123	43.93%
Psychological/spiritual		
Continue care or monitor (0–1)	239	85.97%
Need further management (2–3)	39	14.03%
Missing data	2	0.72%
Family/carer		
Continue care or monitor (0–1)	168	60.22%
Need further management (2–3)	111	39.78%
Missing data	1	0.36%
Phase		
Phase 1~3	133	47.50%
Phase 4	147	52.50%

^1^ PCOC: Palliative Care Outcome Collaboration. ^2^ SAS: Symptom Assessment Scale; Each symptom was rated on a scale of 0 (absent) to 10 (worst possible) points. ^3^ AKPS: Australia-modified Karnofsky Performance Status; Physical function was rated from 0 (death) to 100 (normal physical function). ^4^ RUG-ADL: Resource Utilization Groups–Activities of Daily Living; Three-point item (1–independent or supervision only; 3–total dependence/tube fed) was rated in eating domain, and the other three activities were based on five-point items (1–independent; 5–two or more persons assist); Total scores was from 4 to 18 points. ^5^ PCPSS: Palliative Care Problem Severity Score.

**Table 2:** Characteristics of Patients between Non-terminal and Terminal Phase.

Characteristics	Phase 1–3 N/% Mean ± SD	Phase 4 N/% Mean ± SD	*p*-Value
Total patients	133	147	
Age at death (years)	67.45 ± 16.42	67.97 ± 15.42	0.820
Gender			0.567
Male	85 (63.91%)	88 (59.86%)	
Female	48 (36.09%)	59 (40.14%)	
Types of palliative care services			0.359
Inpatient palliative care unit	62 (46.62%)	69 (47.73%)	
alliative care consultation service	36 (27.07%)	48 (32.88%)	
Palliative home care service	33 (24.81%)	29 (19.86%)	
Reasons of discharge			0.039
Death	108 (81.20%)	133 (90.48%)	
Impending death discharge	25 (18.80%)	14 (9.52%)	
Diagnosis			0.792
Cancer	112 (84.21%)	121 (82.31%)	
Non-cancer	21 (15.79%)	26 (17.69%)	
Metastatis present			0.615
Yes	83 (85.57%)	96 (88.89%)	
No	14 (14.43%)	12 (11.11%)	
Duration of PCOC ^1^ evaluation (days)	12.75 ± 18.29	14.79 ± 20.92	0.847
SAS ^2^			
Distress from difficulty sleeping	2.72 ± 2.82	2.90 ± 3.38	0.665
Distress from appetite	2.54 ± 3.14	2.75±3.54	0.665
Distress from nausea	0.80±1.90	0.72±1.79	0.693
Distress from bowels	1.62 ± 2.67	1.77 ± 2.86	0.333
Distress from breathing	3.56 ± 3.14	4.63 ± 3.49	0.936
Distress from fatigue	3.86 ± 3.26	4.02 ± 3.74	0.014
Distress from pain	2.82 ± 2.74	2.21 ± 3.07	0.926
Total scores	17.93 ± 13.44	18.9816.02	0.009
AKPS ^3^	29.32 ± 13.10	17.01 ± 7.25	0.947
RUG-ADL ^4^	14.98 ± 2.33	16.52 ± 1.77	<0.0001
PCPSS ^5^			
Pain			0.009
Continue care or monitor (0–1)	102 (76.69%)	131 (89.12%)	
Need further management (2–3)	31 (23.31%)	16 (10.88%)	
Other symptoms			0.015
Continue care or monitor (0–1)	64 (48.12%)	93 (63.27%)	
Need further management (2–3)	69 (51.88%)	54 (36.73%)	
Psychological/spiritual			0.184
Continue care or monitor (0–1)	110 (82.71%)	129 (88.97%)	
Need further management (2–3)	23 (17.29%)	16 (11.03%)	
Family/carer			0.621
Continue care or monitor (0–1)	82 (62.12%)	86 (58.50%)	
Need further management (2–3)	50 (37.88%)	61 (41.50%)	

^1^ PCOC: Palliative Care Outcome Collaboration. ^2^ SAS: Symptom Assessment Scale; Each symptom was rated on a scale of 0 (absent) to 10 (worst possible) points. ^3^ AKPS: Australia-modified Karnofsky performance status; Physical function was rated from 0 (death) to 100 (normal physical function). ^4^ RUG-ADL: Resource Utilization Groups–Activities of Daily Living; Three-point item (1–independent or supervision only; 3–total dependence/tube fed) was rated in eating domain, and the other three activities were based on five-point items (1–independent; 5–two or more persons assist); Total scores was from 4 to 18 points. ^5^ PCPSS: Palliative Care Problem Severity Score.

**Table 3:** Univariate Analysis Exploring Risk Factors of Unexpected Death in Palliative Care Settings.

Characteristics	Odds Ratio	95% CI		*p* Value
Age at death	0.99	0.98–1.01		0.783
Gender				
Male	Reference			
Female	0.84	0.52–1.37		0.487
Types of palliative care services				
Inpatient palliative care unit	Reference			
Palliative care consultation service	0.84	0.48–1.45		0.521
Palliative home care service	1.34	0.74–2.45		0.335
Reasons of discharge				
Death	Reference			
Impending death discharge	2.20	1.09–4.44		0.028
Diagnosis				
Non-cancer	Reference			
Cancer	1.15	0.61–2.15		0.672
Metastatis present				
No	Reference			
Yes	0.74	0.33–1.69		0.476
Duration of PCOC ^1^ evaluation	0.99	0.98–1.01		0.391
SAS ^2^				
Distress from difficulty sleeping	0.98	0.90–1.07		0.653
Distress from appetite	0.98	0.91–1.06		0.638
Distress from nausea	1.03	0.89–1.18		0.728
Distress from bowels	0.98	0.89–1.08		0.68
Distress from breathing	0.91	0.84–0.98		0.015
Distress from fatigue	0.99	0.92–1.06		0.731
Distress from pain	1.08	0.98–1.18		0.106
Total scores	0.99	0.98–1.01		0.582
AKPS ^3^	1.13	1.09–1.17		<0.0001
RUG-ADL ^4^	0.65	0.56–0.76		<0.0001
PCPSS ^5^				
Pain				
Continue care or monitor (0–1)	Reference			
Need further management (2–3)	2.49	1.29–4.80		0.007
Other symptoms				
Continue care or monitor (0–1)	Reference			
Need further management (2–3)	1.86	1.15–2.99		0.011
Psychological/spiritual				
Continue care or monitor (0–1)	Reference			
Need further management (2–3)	1.69	0.85–3.35		0.136
Family/carer				
Continue care or monitor (0–1)	Reference			
Need further management (2–3)	0.86	0.53–1.39		0.538

^1^ PCOC: Palliative Care Outcome Collaboration.^2^ SAS: Symptom Assessment Scale. ^3^ AKPS: Australia-modified Karnofsky Performance Status.^4^ RUG-ADL: Resource Utilization Groups–Activities of Daily Living. ^5^ PCPSS: Palliative Care Problem Severity Score.

**Table 4:** Multivariate Analysis Exploring Risk Factors of Unexpected Death in Palliative Care Settings.

Characteristics	Odds Ratio	95% CI		*p* Value
Age at death	1.00	0.98–1.03		0.988
Gender				
Male	Reference			
Female	1.27	0.57–2.83		0.563
Reasons of discharge				
Death	Reference			
Impending death discharge	3.93	1.20–12.94		0.024
SAS ^1^				
Distress from breathing	0.83	0.71–0.97		0.021
Total scores	1.00	0.97–1.04		0.840
AKPS ^2^	1.12	1.10–1.21		<0.0001
RUG-ADL ^3^	0.88	0.71–1.08		0.218
PCPSS ^4^				
Pain				
Continue care or monitor (0–1)	Reference			
Need further management (2–3)	1.32	0.43–4.10		0.630
Other symptoms				
Continue care or monitor (0–1)	Reference			
Need further management (2–3)	1.61	0.73–3.56		0.236

^1^ SAS: Symptom Assessment Scale. ^2^ AKPS: Australia-modified Karnofsky Performance Status. ^3^ RUG-ADL: Resource Utilization Groups–Activities of Daily Living. ^4^ PCPSS: Palliative Care Problem Severity Score.

**Table 5:** Subgroup Analysis Exploring Risk Factors of Unexpected Death among Patients with Different AKPS.

Characteristics	AKPS ≥ 50				AKPS < 50			
	Odds Ratio	95% CI	*p* Value		Odds Ratio	95% CI	*p* Value	
Age at death	1.01	0.96–1.07	0.582		1.00	0.98–1.02	0.974	
Gender								
Male	Reference				Reference			
Female	0.39	0.09–1.79	0.227		1.19	0.62–2.27	0.610	
SAS ^1^								
Distress from breathing	0.95	0.76–1.20	0.664		0.89	0.80–0.99	0.024	
Distress from appetite	1.10	0.84–1.46	0.487		0.89	0.80–0.99	0.035	
RUG-ADL ^2^	0.66	0.44–0.99	0.045		0.69	0.57–0.83	0.0001	
Pain								
Continue care or monitor (0–1)	Reference				Reference			
Need further management (2–3)	2.25	0.12–43.84	0.592		1.59	0.67–3.78	0.295	
Other symptoms								
Continue care or monitor (0–1)	Reference				Reference			
Need further management (2–3)	1.79	0.40–7.95	0.442		2.35	1.16–4.76	0.018	

^1^ SAS: Symptom Assessment Scale. ^2^ RUG-ADL: Resource Utilization Groups–Activities of Daily Living.

## Data Availability

The datasets used and analyzed during the current study are not publicity available, but are available from the corresponding author upon reasonable request with the permission of Taichung Veterans General Hospital, Taiwan.
